# Adaptation to chronic drought modifies soil microbial community responses to phytohormones

**DOI:** 10.1038/s42003-021-02037-w

**Published:** 2021-05-03

**Authors:** Emma J. Sayer, John A. Crawford, James Edgerley, Andrew P. Askew, Christoph Z. Hahn, Raj Whitlock, Ian C. Dodd

**Affiliations:** 1 Lancaster Environment Centre, Lancaster University, Lancaster, UK; 2 Department of Evolution, Ecology and Behaviour, University of Liverpool, Liverpool, UK

**Keywords:** Ecosystem ecology, Climate-change ecology

## Abstract

Drought imposes stress on plants and associated soil microbes, inducing coordinated adaptive responses, which can involve plant–soil signalling via phytohormones. However, we know little about how microbial communities respond to phytohormones, or how these responses are shaped by chronic (long-term) drought. Here, we added three phytohormones (abscisic acid, 1-aminocyclopropane-1-carboxylic acid, and jasmonic acid) to soils from long-term (25-year), field-based climate treatments to test the hypothesis that chronic drought alters soil microbial community responses to plant stress signalling. Phytohormone addition increased soil respiration, but this effect was stronger in irrigated than in droughted soils and increased soil respiration at low phytohormone concentrations could not be explained by their use as substrate. Thus, we show that drought adaptation within soil microbial communities modifies their responses to phytohormone inputs. Furthermore, distinct phytohormone-induced shifts in microbial functional groups in droughted vs. irrigated soils might suggest that drought-adapted soil microorganisms perceive phytohormones as stress-signals, allowing them to anticipate impending drought.

## Abstract (web summary)

Emma Sayer et al. use a 25-year field experiment to investigate how microbial community responses to phytohormones are affected by drought. Phytohormone-induced shifts in microbial functional groups suggest that drought adaptation within soil microbial communities mediates responses to plant stress signalling.

## Introduction

Interactions between plants and soil microorganisms play a critical role in determining the response of terrestrial ecosystems to a changing climate^1^. The establishment and maintenance of relationships between plants and microbes requires mutual recognition of the responses of both partners to changes in their immediate environments^2,3^, which is largely thought to be mediated by the reciprocal exchange of resources^4^. Plants can rapidly stimulate microbial activity via root exudates and signalling molecules, such as phytohormones^5^, and plants adapted to different climate conditions can modify soil microbial activity by altering the composition of root exudates^6^. Whereas most root inputs function largely as a source of carbon and nutrients for soil microbial communities, phytohormones can act both as a substrate and as signalling molecules^2^. Molecular signalling represents an important communication pathway between plants and microbes, whereby plant hormone inputs modify microbial community structure or activity, and microbial metabolism or synthesis of phytohormones enables them to influence plant growth and performance^7–9^. Such bidirectional communication between plants and microorganisms can result in coordinated responses to environmental changes^9,10^, which will shape ecosystem function. Phytohormones can be released into the soil via diffusion or active transport, as well as actively exuded by roots^11,12^, and the root exudates of drought-stressed plants show increased concentrations of phytohormones^13^. Whereas numerous studies have investigated soil microbial responses to root exudate compounds such as sugars, amino acids, organic acids^14,15^ or secondary metabolites^16^, we still know very little about the influence of phytohormones on soil microbial activity.

The role of phytohormones in coordinating plant-microbial responses to drought is of particular interest, because plants are immobile and their survival depends largely upon their ability to rapidly adjust their physiology and growth to mitigate the impacts of drought stress, processes which are usually mediated by phytohormones^3,17^. Roots in contact with drying soil show altered hormone accumulation^18–20^ and increased hormone efflux to the rhizosphere can shape plant-associated microbial communities, which in turn influence plant performance, resulting in coordinated plant-microbial responses to drought^21,22^. Soil microbial community composition is altered by exogenous application of several phytohormones involved in plant drought responses, including abscisic acid (ABA), ethylene and jasmonates^23^. ABA is an important signalling molecule in plants and microorganisms^24^. ABA is synthesized throughout the plant in response to decreased tissue water status and maintains root growth and hydraulic conductance in drying soil^7^. Water-stressed roots exude ABA into the soil^25^ where it can be metabolized by some fungi and bacteria^26^. Drought also stimulates plant production of ethylene and its precursor 1-aminocyclopropane-1-carboxylic acid (ACC). As ACC usually inhibits root elongation^27^, bacterial degradation of ACC can stimulate root growth, even under water stress^17^. Finally, although jasmonates such as jasmonic acid (JA) are usually associated with biotic stress^28^, they also affect plant drought responses by modulating root hydraulic conductance and stomatal closure^29^. Jasmonates can influence rhizosphere microbial communities indirectly by altering the concentrations of different compounds in root exudates^30^ and marked shifts in microbial community structure have also been observed in direct response to exogenous application of methyl jasmonate^23^. Hence, by modulating plant stress responses and shaping microbial community structure, phytohormones could play a significant role in the co-adaptation of plants and soil microbial communities to drought^9^.

Research into plant hormone-mediated microbial activity under drought conditions has largely focussed on rhizosphere or endophytic organisms that are tightly associated with the plant^31–33^. It is well known that plant growth-promoting rhizobacteria can interact with or manipulate plant hormone signalling by synthesizing or metabolizing phytohormones^7,8^. However, many other naturally occurring soil organisms respond to root efflux of phytohormones^2^, but the role of phytohormones in shaping soil microbial communities and processes more generally has received little attention. As plant drought responses are mediated by phytohormones, frequent or chronic water deficit could result in high exposure of soil microbial communities to these stress-signalling molecules. The responses of broad soil microbial communities to root phytohormone efflux from water-stressed plants represent a major gap in our understanding of plant–soil interactions under drought, because numerous important ecosystem processes are modulated by microbial community structure and activity in bulk soils^1^. Shifts in the relative abundance and dominance of soil microorganisms in response to drought can be regarded as community-level adaptation, resulting in differentiated microbial communities in droughted vs. non-droughted soils^10^, which in turn can modify microbially mediated soil processes^34^. In particular, proliferation of slow-growing oligotrophic microbes under drought can enhance community-level drought tolerance and the accompanying changes in resource-use strategies alter carbon and nutrient dynamics in soils^10,35^. Hence, identifying the role of plant stress hormones in shaping microbial communities and activities under drought is an important first step to establish whether molecular signalling enables plant-microbial co-adaptation, which could ultimately shape ecosystem processes.

Here we assessed the effects of three drought-associated phytohormones on soil microbial activity and community structure, to investigate whether chronic drought and irrigation treatments influence soil microbial community responses to phytohormones. We used soils from chronic drought and irrigation treatments within the Buxton Climate Change Impacts Study, where temperature and rainfall have been experimentally manipulated since 1993^36^. Such prolonged treatments (25 years) represent a strong selective pressure on both plants and soil microbial communities^37–39^. Previous work at the study site demonstrated that drought-mediated changes in soil microbial communities were linked to changes in the plant community via shifts in plant traits representing altered resource quality for soil microorganisms^37,38,40^. Hence, chronic climate treatments have altered soil microbial community structure and we hypothesized that soil microbial communities from long-term drought plots would respond more strongly to plant stress hormones, compared with soil microbes from irrigated or control plots. To test our hypotheses, we quantified changes in soil microbial activity (respiration rates) in response to different concentrations of ABA, JA and ACC using a microplate assay, and we assessed changes in soil microbial community structure using phospholipid fatty acid (PLFA) biomarkers. We observed increased soil microbial respiration following phytohormone additions, which could not be explained by substrate use alone. Importantly, the magnitude of the respiration response differed among climate treatments and shifts in microbial biomarkers with phytohormone addition differed markedly between soils subjected to long-term drought or irrigation treatments. Thus, our study demonstrates that adaptation to long-term drought modifies the responses of soil microbial communities to plant stress hormones.

## Results

### Soil respiration in response to phytohormone additions

**Fig. 1:**
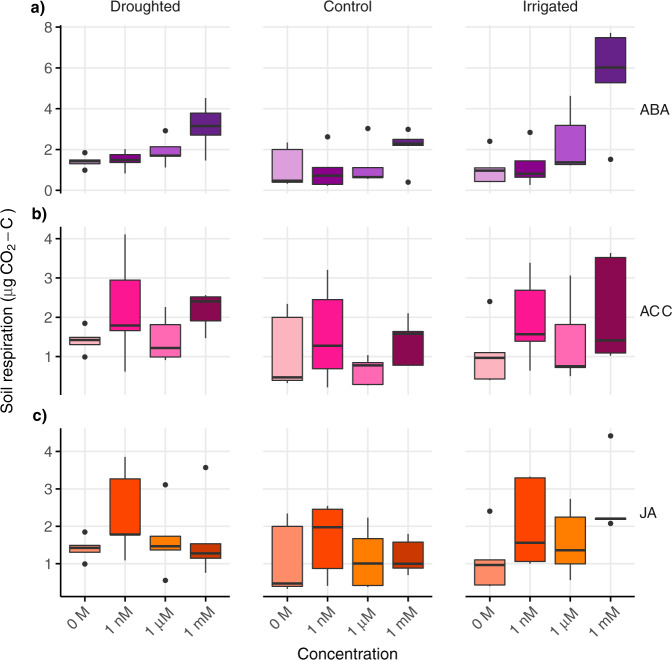
Respiration rates (CO_2_ efflux) following phytohormone additions to soils from long-term climate change treatments. Soils collected from control, droughted and irrigated plots in the Buxton Climate Change Impacts Study were incubated for 6 h with addition of **a** abscisic acid (ABA), **b** 1-aminocyclopropane-1-carboxylic acid (ACC) or **c** jasmonic acid (JA) at three concentrations compared to procedural controls (concentration = 0 M); boxes denote the 25th and 75th percentiles, and median lines are given for *n* = 5 replicates based on independent field plots; whiskers indicate values up to 1.5× the interquartile range and dots indicate outliers.

Respiration rates in procedural controls without phytohormone addition did not differ among climate treatments and the soils contained similar concentrations of extractable carbon (C) and nitrogen (N; Table 1). However, the respiration rate of all soils increased following phytohormone addition and the magnitude of the response differed among climate treatments, whereby the increases in respiration were generally greater in irrigated soils compared to droughted or control soils (Fig. 1). There was no clear trend in respiration responses with increasing phytohormone concentration, but we note that the extra carbon released by increased respiration at nanomolar phytohormone concentrations exceeded the amount of carbon added with the phytohormone solutions (Supplementary Methods 1 and Table S1), indicating that microbial activity was triggered by phytohormone inputs at nanomolar concentrations. Furthermore, the effects of phytohormones on soil respiration persisted when they were added in conjunction with root exudate solution (Supplementary Methods 2 and Fig. S1).

**Table 1:** Soil properties and microbial biomarker groups in soils from chronic climate change treatments.

	Droughted	Control	Irrigated
Soil water content (%)	32.6 ± 1.1^a^	32.7 ± 1.2^a^	41.6 ± 3.3^b^
Extractable soil C (mg g^−1^)	282 ± 20	313 ± 29	325 ± 27
Extractable soil N (mg g^−1^)	48.6 ± 4.7	54.4 ± 7.2	46.8 ± 3.5
Total PLFA biomass (nM g^−1^)	549 ± 39^a^	601 ± 54^a^	725 ± 48^b^
Fungal biomass (nM g^−1^)	80 ± 5.7^b^	101 ± 7.1^a^	125 ± 7.3^c^
G− biomarkers (%)	45 ± 0.4	45 ± 0.5	46 ± 0.4
G+ biomarkers (%)	22 ± 0.4	22 ± 0.6	22 ± 0.2
Fungal biomarkers (%)	14 ± 1.1	14 ± 0.9	14 ± 0.8
AMF biomarkers (%)	4.5 ± 0.1	4.5 ± 0.1	4.6 ± 0.1
Actinomycete biomarkers (%)	11.8 ± 0.6	11.6 ± 0.6	11.4 ± 0.5
G+ : G− ratio	0.48 ± 0.01	0.48 ± 0.01	0.47 ± 0.01
F : B ratio	0.26 ± 0.004^b^	0.32 ± 0.01^a^	0.32 ± 0.01^a^

Soil water content, extractable soil carbon (C) and nitrogen (N), active microbial biomass derived from phospholipid fatty acid (PLFA) analysis (Total PLFA biomass) and PLFA biomarkers representing microbial functional groups in soils collected from droughted, irrigated and control plots in the Buxton Climate Change Impacts Study; G+ is Gram-positive bacteria, G− is Gram-negative bacteria, F : B is the ratio of fungal to bacterial biomarkers and AMF is arbuscular mycorrhizal fungi; means ± SEs are given for *n* = 5 replicates per climate treatment and different superscript letters denote significant differences among treatments at *p* < 0.05.

Following ABA addition, respiration increased only at the highest concentration (1 mM) and the increase differed among climate treatments (climate × concentration interaction: *χ*^2^ = 58.9, *p* < 0.001; Fig. 1a), with a significantly greater respiration response in the irrigated soils than in the controls (*p* < 0.001) and droughted soils (*p* = 0.001). Although the increase in respiration from the control soils was not significant (*d* = 0.8; *p* = 0.11), respiration from droughted soils doubled in response to the highest concentration of ABA (*d* = 1.95; *p* = 0.006) and increased more than fivefold in the irrigated soils (*d* = 2.27; *p* < 0.001). Hence, the respiration response to ABA was strongest in irrigated soils.

ACC addition also stimulated respiration and the response at different concentrations was similar among treatments (climate + concentration effect; *χ*^2^ = 18.1, *p* = 0.003; Fig. 1b). Respiration rates increased at ACC concentrations of 1 mM (*p* = 0.015) and 1 nM (*p* = 0.013), but not at 1 μM. The increase in respiration from the control soils in response to ACC was negligible (*d* < 0.3), whereas respiration from droughted soils was 1.5× higher than the procedural controls (*d* = 1.42 and *d* = 0.80 for 1 mM and 1 nM, respectively) and respiration from the irrigated soils was twice as high (*d* = 1.10 and *d* = 0.99 for 1 mM and 1 nM, respectively). Thus, soil respiration showed an unusual response to ACC addition, which was more pronounced in irrigated soils.

Both high and low concentrations of JA were associated with increased respiration rates, but the intermediate concentration was not (climate + concentration effect: *χ*^2^ = 13.5, *p* = 0.019; Fig. 1c). Surprisingly, the lowest concentration of JA (1 nM) had the largest effect on respiration in droughted and control soils (*p* = 0.006, *d* = 0.5, 0.1 and 0.11 for control, droughted and irrigated soils, respectively), whereas the increase in respiration at the highest concentration was only apparent in the irrigated soils (1 mM, *p* = 0.033, *d* = 3.1) and there was no effect at the intermediate concentration of JA (1 μM). Thus, the lowest concentration of JA stimulated respiration regardless of climate treatment.

### Shifts in biomarker functional groups

Total pre-incubation PLFA biomass was 21% higher in the irrigated plots compared to the controls and 32% higher than the droughted plots (*F*_2,8_ = 11.2, *p* = 0.005; Table 1). Total biomass increased in all soils during incubation but was unaffected by phytohormone addition. Actinomycete biomarkers did not differ among climate treatments, did not change during the incubation and were also unaffected by phytohormone addition. However, we observed changes in the relative abundance of biomarker functional groups in response to phytohormone addition, which differed among climate treatments, indicated by significant hormone × climate interactions for biomarkers representing Gram-positive bacteria (*χ*^2^ = 25.6, *p* = 0.002), Gram-negative bacteria (*χ*^2^ = 31.2, *p* = 0.001), the ratio of Gram-positive to Gram-negative biomarkers (G+ : G− ratio; *χ*^2^ = 32.2, *p* < 0.001), saprophytic fungi (*χ*^2^ = 42.1, *p* < 0.001) and arbuscular mycorrhizal (AM) fungi (*χ*^2^ = 53.6, *p* < 0.001).

**Fig. 2:**
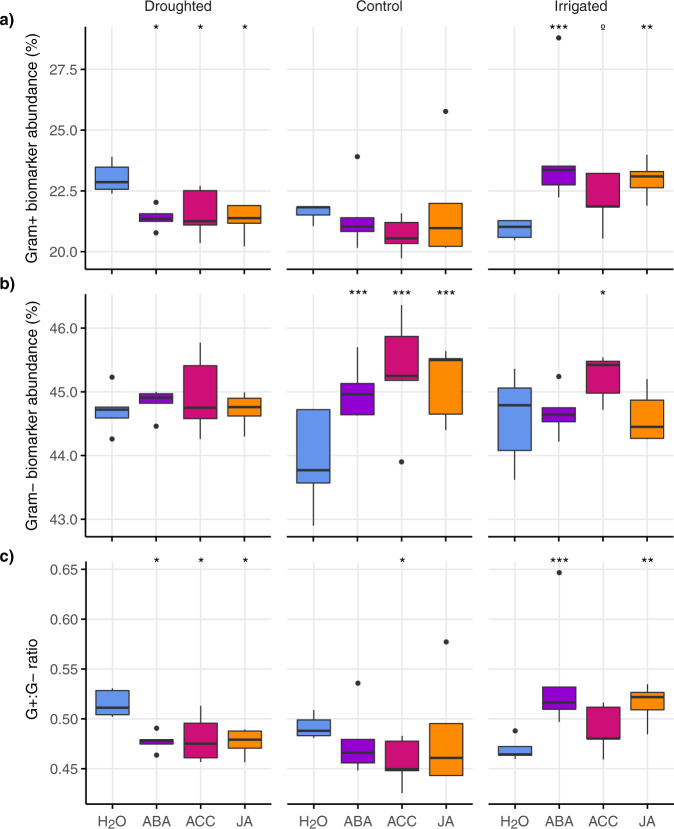
Bacterial biomarker functional groups following phytohormone additions to soils from long-term climate change treatments. Relative abundances of **a** Gram-positive (Gram+) and **b** Gram-negative (Gram−) bacteria, and **c** the ratio of Gram-positive to Gram-negative bacteria (G+ : G− ratio) measured in soils collected from control, droughted and irrigated plots in the Buxton Climate Change Impacts Study, after incubation for 24 h with abscisic acid (ABA), 1-aminocyclopropane-1-carboxylic acid (ACC), jasmonic acid (JA), or water (procedural controls; H_2_O); boxes denote the 25th and 75th percentiles, and median lines are given for *n* = 5 replicates based on independent field plots; whiskers indicate values up to 1.5× the interquartile range and dots indicate outliers; effects of hormone application within climate treatments are shown as ***p < 0.001; **p < 0.01, *p < 0.05 and ^o^ for trends at *p* < 0.1.

The relative abundance of Gram-positive and Gram-negative biomarkers did not differ among treatments before incubation (Table 1) but responded differently to phytohormone additions. The abundance of Gram-positive biomarkers remained unchanged in control soils but increased significantly in irrigated soils with the addition of ABA and JA, and there was a trend towards increased Gram-positive biomarkers with ACC. By contrast, in droughted soils Gram-positive biomarker abundance declined significantly with the addition of all three hormones (Fig. 2a). Gram-negative biomarker abundance remained unchanged in droughted soils but increased with ABA, ACC and JA addition to control soils, and with ACC addition to irrigated soils (Fig. 2b). Thus, in response to phytohormone addition, the relative abundance of Gram-positive biomarkers increased in irrigated soils but declined in droughted soils.

The G+ : G− ratio before incubation was similar among climate treatments (Table 1) but largely followed the response of Gram-positive biomarkers to incubation with phytohormones. The G+ : G− ratio increased with addition of ABA and JA in irrigated soils, but declined in control soils with ACC addition and declined in droughted soils in response to all hormones (Fig. 2c).

Fungal biomass before incubation was 21% lower in droughted plots and 24% higher in the irrigated plots compared to the controls (*F*_6,8_ = 19.03, *p* < 0.001; Table 1). Fungal biomass increased in all climate treatments during incubation but there was no effect of phytohormone addition. Similarly, the ratio of fungi to bacteria (F : B ratio) before incubation was lower in the droughted plots than in the irrigated plots or controls (*F*_2,8_ = 12.6, *p* = 0.003; Table 1) but phytohormone addition did not affect the F : B ratio in any climate treatment.

**Fig. 3:**
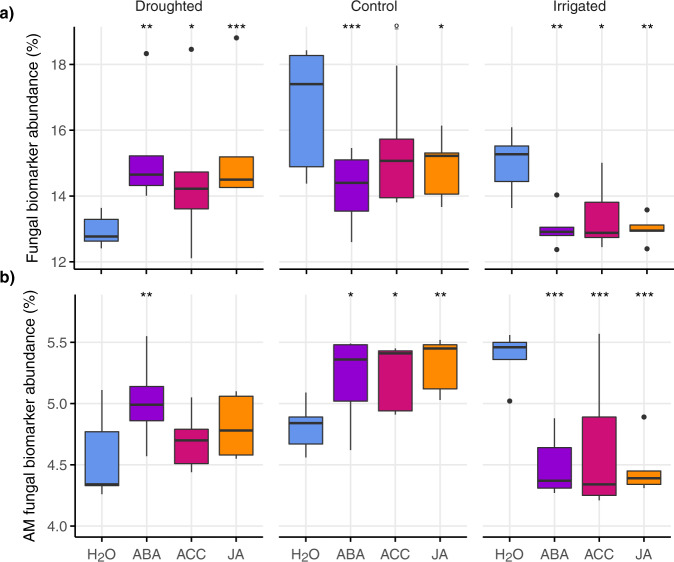
Fungal biomarker functional groups following phytohormone additions to soils from long-term climate change treatments. Relative abundances of **a** saprophytic fungi and **b** arbuscular mycorrhizal (AM) fungi measured in soils collected from control, droughted and irrigated plots in the Buxton Climate Change Impacts Study after incubation for 24 h with abscisic acid (ABA), 1-aminocyclopropane-1-carboxylic acid (ACC), jasmonic acid (JA), or water (procedural controls; H_2_O); boxes denote the 25th and 75th percentiles, and median lines are given for *n* = 5 replicates based on independent field plots; whiskers indicate values up to 1.5× the interquartile range and dots indicate outliers; effects of hormone application within climate treatments are shown as ***p < 0.001; **p < 0.01, *p < 0.05 and ^o^ for trends at *p* < 0.1.

The abundance of saprophytic fungal biomarkers before incubation was similar among treatments (Table 1) but declined significantly in control soils in response to ABA and JA, and there was a trend towards a decline in fungal biomarkers with ACC addition. The decline in saprophytic fungal biomarkers with phytohormone addition was even stronger in irrigated soils, with significantly lower abundance in response to all three hormone treatments. By contrast, saprophytic fungal biomarker abundance in the droughted soils increased in response to all three hormone treatments (Fig. 3a). Thus, in response to phytohormone addition, the relative abundance of saprophytic fungal biomarkers declined in irrigated soils but increased in droughted soils. The abundance of AM fungal biomarkers did not differ among treatments before incubation (Table 1) but AM fungal biomarkers increased significantly in the control soils with the addition of all three hormones and in droughted soils with ABA addition. By contrast, addition of all three hormones significantly reduced AM fungal biomarker abundance in the irrigated soils. Thus, in response to phytohormone addition, the relative abundance of AM fungal biomarkers increased in the control and droughted soils but declined in the irrigated soils (Fig. 3b).

## Discussion

Our study demonstrates that community-level adaptation to chronic climate change modifies the response of soil microbes to phytohormone inputs. Phytohormone addition clearly stimulated soil microbial activity, but the magnitude of the effects differed for soil microbial communities conditioned under different climate treatments at Buxton. The increased respiration rates at low concentrations of phytohormones and the contrasting shifts in microbial functional groups in droughted vs. irrigated soils are particularly intriguing. Here we explore the possibility that soil microbial communities might respond to phytohormones as signals of impending water stress rather than as substrates for growth.

Increased respiration rates in response to phytohormones could indicate that bulk soil microorganisms can utilize all three hormones as substrates, and the greater respiration response to phytohormones in the irrigated compared to droughted soils strongly suggests community-level adaptation of soil microorganisms to field conditions. Microbial community-level adaptation to drought often involves shifts towards organisms with greater tolerance to water deficit, as well as increased abundance of slow-growing taxa with conservative resource-use strategies^10,41^. Distinct resource-use efficiencies of the microbial communities would explain some of the differences in the respiration responses to phytohormones, despite similar concentrations of readily available soil C and N among climate treatments (Table 1). In the irrigated soils, the substantial increases in respiration following phytohormone addition are characteristic of a microbial community dominated by copiotrophic organisms that preferentially use labile substrate and can rapidly respond to changes in resource inputs^10^. In droughted soils, smaller respiration responses to phytohormones correspond to a stress-tolerant microbial community dominated by taxa with reduced metabolic capacity^41^. By contrast, the control plots at our study site experience large natural fluctuations in soil moisture^38^ resulting in microbial communities composed of taxa with broad tolerances^34^, which is reflected in the variable respiration rates in control soils, both with and without phytohormone addition (Fig. 1). Hence, highly differentiated microbial communities in soils sampled several months after the end of the summer drought treatment at Buxton (Supplementary Methods 3 and Fig. S2) indicate lasting shifts in microbial community structure that likely underpin the distinct respiration responses in our experiment.

Three lines of evidence indicate that microbial utilization of phytohormones as a substrate cannot fully explain our findings. First, if increased respiration rates were solely due to microbial utilization of phytohormones as substrates, we would expect the highest respiration rates at the highest phytohormone concentrations in all climate treatments, but this was only observed for ABA (Fig. 1a). Thus, the consistent response to ABA at high concentrations likely reflects microbial utilization of ABA as the substrate^26^. However, the inconsistent response to ACC addition is surprising, because numerous microorganisms can also utilize ACC as a source of C and N^7^. Second, the effects of phytohormones on soil respiration persisted even when they were added in conjunction with root exudate solution, which provides an ample source of C and N to soil microbes (Supplementary Table S2 and Fig. S1). Finally, microbial use of phytohormones as substrate cannot explain the increased respiration at the lowest concentrations of JA and ACC, where the additional release of C as CO_2_ exceeded C inputs (Supplementary Methods 1 and Table S1). Such a disproportionate increase in respiration in response to very small resource inputs indicates that JA and ACC stimulated microbial mineralization of existing soil C, a phenomenon previously demonstrated for other organic substances contained in root exudates^14,15,42,43^. Hence, whereas increased respiration at high (millimolar) concentrations of all phytohormones likely reflects their utilization by microbes as C and nutrient sources, increased respiration following ACC and JA addition at nanomolar concentrations suggests that these phytohormones can also trigger microbial activity and the extent of the response clearly differed among climate treatments. Whereas increased respiration with 1 nM ACC might be explained by promotion of fungal development at low concentrations (<1 mM)^44^, the respiration response of droughted and irrigated soils to 1 nM JA is particularly intriguing, because plant development appears to benefit more at low concentrations of jasmonates than at high concentrations^45^ but the microbial metabolic pathways for JA are largely unknown^46^. Although it is conceivable that increased respiration at low phytohormone concentrations could be entirely explained by ‘priming’ of soil C^42^, such priming did not occur in response to adding a standard root exudate solution and respiration rates with the root exudate solution did not differ among climate treatments (Supplementary Methods 2 and Fig. S1). Hence, although we are unable to identify mechanisms, we propose that climate-driven adaptation of soil microbial communities not only alters their capacity to utilize phytohormones as a substrate but might also modulate their responses to plant stress hormones as signalling molecules.

The contrasting effects of phytohormones on biomarker abundances in droughted vs. irrigated soils provide further strong evidence for community-level microbial adaptation to drought. As microbial biomass was unaffected by phytohormone addition, the observed changes in the relative abundances of biomarker functional groups in response to phytohormones likely represent shifts in dominance, activity, or turnover, rather than growth in overall community size^47^. The disparate response of fungal biomarkers in droughted vs. irrigated soils is particularly striking (Fig. 3), because fungi are inherently more drought-resistant than bacteria^41,48^, whereas bacteria grow faster and outcompete fungi for access to labile substrates^49^ such as phytohormones. However, if chronic drought has selected against fast-growing stress-intolerant bacteria^10^, a greater proportion of resources might be available to fungi. Previous work at our study site demonstrated large changes in fungal community structure and the absence of ca. 25% of fungal taxa in the droughted plots^38^, as well as shifts in mycorrhizal colonization rates and extraradical hyphal abundance^50^, suggesting that this chronic treatment has selected against drought-sensitive fungi. A more stress-tolerant fungal community in droughted soils, combined with resource competition between drought-adapted fungi and bacteria could explain why fungal abundance increased following phytohormone application in droughted soils, but declined in the controls and irrigated soils.

Many Gram-negative bacteria are fast-growing organisms^51^ and a wide range of Gram-negative genera can metabolize ACC^7,52^, so it is noteworthy that the largest increases in Gram-negative biomarker abundance occurred after adding ACC to irrigated and control soils. Conversely, selection against fast-growing opportunistic bacteria would explain why Gram-negative bacteria did not respond to any hormone additions in the droughted soils, as chronic drought would favour stress-tolerant, slow-growing Gram-negative taxa with thick cell walls that are more similar to Gram-positive physiology^10,53,54^. At first glance, it seems surprising that the abundance of Gram-positive biomarkers in the droughted soils declined following phytohormone addition (Fig. 2a), because they are generally assumed to be more stress-tolerant than Gram-negative bacteria^41,48^. However, we speculate that the declines in Gram-positive biomarker abundance following addition of all three phytohormones indicate drought-adapted bacteria that respond negatively to stress signalling, possibly by investing resources in survival strategies such as dormancy, osmolytes or spore production instead of growth and turnover^41,48,55^.

The contrasting responses of Gram-positive biomarker abundance following phytohormone additions to droughted vs. irrigated soils raise the intriguing possibility that drought-adapted soil microbial communities might perceive these phytohormones as signals of impending water stress rather than as a substrate for growth. Phytohormones could act as early-warning signals for soil microbial communities, because root hormone accumulation in relatively moist soil (Ψ < −0.1 MPa^56^) indicates that plants detect soil water deficits at much higher bulk soil water potentials than bacteria (Ψ < −1.0 MPa^57^). If phytohormones activate acquired microbial stress responses, they would allow microbes to improve defences against future stress^58^. As drought stress responses such as spore formation or solute accumulation are energetically costly^41^, it is possible that some of the increased respiration in response to phytohormones in the droughted soils reflects enhanced metabolic demands of microorganisms as they activate stress responses^58,59^. However, we note that although increased respiration in soils from different climate treatments might be attributed to distinct mechanisms, our measurements cannot distinguish between substrate-induced respiration and respiration as a result of other microbial metabolic activities. Hence, we propose an emerging hypothesis, whereby phytohormones induce adaptive microbial responses or defences to impending water stress and chronic drought selects for microorganisms that respond to stress signalling by entering dormancy, or producing spores or extracellular polymeric substances, rather than increasing growth and abundance^41,48,55^. Clearly, our experiment cannot identify the metabolic pathways involved in microbial responses to plant stress signalling. However, our findings highlight an intriguing new avenue for research to identify the mechanisms underpinning coordinated plant-microbial responses to drought.

In summary, we demonstrate that chronic drought modifies the response of soil microbial communities to phytohormones associated with plant water deficit. Differences in the structure and metabolic activity of the soil microbial communities in droughted vs. irrigated soils suggest community-level adaptation to the long-term climate treatments, which shaped the response of the microbial communities to phytohormone additions. Given the divergent responses of both microbial activity and biomarker abundances in droughted vs. irrigated soils, we call for targeted studies to address our hypothesis that drought-adapted microbial communities might use phytohormones as signals of impending water stress. The capacity of soil microbial communities to respond to plant stress-signalling by modulating their metabolic activity could affect numerous microbial processes underpinning ecosystem nutrient and carbon dynamics. Consequently, understanding how phytohormones mediate plant-microbial responses to stress could provide new opportunities to enhance the resistance of ecosystem processes to drought. Although we measured respiration rates as a key response of the soil microbial community, adaptation to drought is often characterized by increased abundance of specialist organisms^10^, which could affect other important soil processes or influence plant performance. Our findings therefore represent an important step towards identifying how root phytohormone efflux by plants drives selection for drought-resistant soil microbial communities, which will advance our understanding of ecosystem responses to climate change.

## Methods

### Study system and field sampling

The Buxton Climate Change Impacts Study (henceforth ‘Buxton’) is located on calcareous grassland in Derbyshire, UK. Climate treatments have been applied to 3 m × 3 m plots in five fully randomized blocks since 1993^36,60^. We sampled soils from three climate treatments: summer drought, in which rainfall is excluded using automated shelters from July–August (‘droughted’), supplemented rainfall to 20% above the long-term average from June–September (‘irrigated’) and control plots that experience the ambient climate (‘controls’). Samples were collected in October 2018, when soil water content in the droughted plots had recovered to control levels (Table 1). Three cores were taken to 10 cm depth in each of the five replicate plots per climate treatment, using a 1 cm diameter punch corer. The cores were homogenized to give one composite sample per plot, sieved (2 mm) to remove stones and debris, and stored at 4 °C for 5 days. We used a 5 g subsample of each soil to determine extractable C and N as a measure of easily available resources to soil microbial communities^61^. The subsamples were extracted in 40 ml 0.5 M potassium sulfate (K_2_SO_4_) solution, shaken for 1 h, filtered through pre-washed filter paper and the total C and N content of the extracts were analysed by oxidation combustion (TOC-L, Shimadzu Corporation, Kyoto, Japan).

### Phytohormone assays

To assess the influence of phytohormones on soil microbial activity, we measured soil respiration using Microresp^TM^, a colorimetric microplate method that measures CO_2_ efflux from a sample via an indicator dye (cresol red) suspended in agar that changes colour when CO_2_ reacts with bicarbonate in the agar gel^62^. Solutions of ABA, ACC and JA were each prepared in concentrations of 1 mM, 1 μM and 1 nM, respectively. Whereas nanomolar concentrations of phytohormones are realistically found in soils^7^, millimolar concentrations are applied in horticulture as plant growth regulators^63,64^ and are relevant as potential substrates for microbes, and 1 μM was included as an intermediate concentration. The phytohormone solutions were compared to procedural controls with deionized water (dH_2_O) only, giving ten phytohormone treatments in total. Three analytical replicates were measured for each sample and phytohormone treatment, giving a total of 450 micro-incubations (5 replicates × 3 climate treatments × 10 hormone treatments × 3 analytical replicates).

For the micro-incubations, soils were brought to 40% field capacity and 0.35 g of soil was added to each 1.2 ml well of a 96-deepwell plate (MicroResp, Aberdeen, UK), which was then pre-incubated at 20 °C for 4 days; samples were fully randomized within each plate. Detection plates (MicroResp, Aberdeen, UK) contained 150 µl 1% agar with 12.5 p.p.m. cresol red indicator dye, 150 mM potassium chloride and 2.5 mM sodium bicarbonate, and initial absorbance was measured on a microplate reader (FLUOstar Omega, BMG Labtech, Ortenberg, Germany) at 570 nm to provide a baseline value^65^. After pre-incubation of the soils, 25 µl aliquots of the phytohormone solutions or dH_2_O were applied and the detection plate was attached using an airtight silicone seal. The plates were incubated for 6 h at 20 °C, after which the colour change in the detection plate was measured at 570 nm to calculate respiration rates (measured absorbance rates minus absorbance for 18 blanks per plate^65^). We determined the calibration curve for absorbance by equilibrating dye solutions at different CO_2_ concentrations prepared with standard gas mixtures (0–5% CO_2_)^65^ and verified the micro-incubations in two trials using soils from the same field plots: Trial 1 was a pilot test of the microplate method to determine appropriate incubation times and indicator gel sensitivity (Supplementary Methods 4 and Figs. S3 and S4), whereas Trial 2 used microcosms with larger quantities of soil and direct measurements of CO_2_ efflux rates (Supplementary Methods 4 and Fig. S5). We further assessed whether the effects of phytohormone addition persisted when added in conjunction with a standard root exudate solution, which represents a readily available source of C and nutrients to soil microbes (Supplementary Methods 2 and Table S2).

### Microbial functional groups

The relative abundance of soil microbial functional groups in the soil samples was determined by PLFA analysis. For each soil sample, six subsamples (5 g fresh weight) were weighed into 50 ml tubes and pre-incubated for 4 days at 20 °C. One subsample was then frozen at −80 °C (*T*_0_ controls) and the other soils received 360 µl of ABA, ACC or JA solution at the highest concentration (1 mM), with dH_2_O as a procedural control, resulting in 60 incubations (3 climate treatments × 4 hormone treatments × 5 replicates). All samples were incubated for 24 h at 20 °C, then frozen at −80 °C before being freeze-dried. PLFAs were extracted from all incubations and the unincubated (*T*_0_) soils using ca. 1 g freeze-dried soil following United States Department of Agriculture protocols^66^ for a high-throughput method^67^. Extracts were analysed by gas chromatography (Agilent Series II 6890, Palo Alto, USA) and peaks were identified using the Sherlock 6.2™ Microbial Identification System (MIDI, Newark, DE, USA).

Our assumption of differentiated microbial communities in the climate treatments was verified by non-metric multidimensional scaling based on Bray–Curtis dissimilarities among PLFA biomarkers in unincubated soils (Supplementary Methods 3 and Fig. S2). The total biomass of all PLFA biomarkers was used as an estimate of active microbial biomass^68^ and PLFA biomarkers representing Gram-positive and Gram-negative bacteria, saprophytic fungi, AM fungi and actinomycetes were used to assess changes in the relative abundances of each functional group (henceforth ‘biomarker functional groups’; Supplementary Methods 5 and Table S3). The ratios of fungal to bacterial biomarkers (F : B ratio) and Gram-positive to Gram-negative biomarkers (G+ : G− ratio) were calculated as additional indicators of change in microbial community structure^69,70^.

### Statistics and reproducibility

Mean values for soil respiration (CO_2_ efflux) were calculated from the three analytical replicates and all analyses were conducted using *n* = 5 replicate plots per climate treatment at Buxton. All data were analysed in R version 3.4.0^71^ using the lme4 package for linear mixed effects models (LMEs^72^). We assessed the effect of phytohormone addition on soil respiration for each hormone with separate LMEs, fitting concentration (1 mM, 1 μM, 1 nM and 0 M), climate treatment and their interaction as fixed effects, and block as a random effect. To compare the magnitude of changes in soil respiration in response to phytohormone additions among climate treatments, we calculated an effect size for each hormone and climate treatment (Cohen’s *d*^73^), representing SDs of difference based on the change in respiration rates relative to the procedural controls. As initial microbial biomass differed among climate treatments (Table 1), we also analysed the effects of phytohormones on soil CO_2_ efflux expressed as the specific respiration rate per unit microbial biomass to indicate differences in microbial metabolic activity^74^, which revealed similar responses to phytohormone additions (Supplementary Methods 6 and Fig. S6).

We assessed differences among treatments in pre-incubation microbial biomass, fungal biomass, microbial biomarkers, and extractable C and N using one-way analyses of variance (*aov* function) with block included as an error term. Changes in microbial functional groups in response to phytohormone addition were then assessed using LMEs for each biomarker group with hormone, climate treatment and their interaction fitted as fixed effects, and block as a random effect.

All LMEs were simplified by sequential removal of terms, comparing models with the Akaike Information Criterion and *p*-values. The best models were compared to appropriate null models using likelihood ratio tests and the final model fit was assessed with diagnostic plots^75^. We give *χ*^2^- and *p*-values for the comparisons against null models for the final model and *p*-values for post hoc treatment contrasts generated using Satterthwaite’s approximation (*difflsmeans* function in the lmerTest package^76^). We report statistically significant effects at *p* < 0.05 and nonsignificant trends at *p* < 0.1.

### Reporting summary

Further information on research design is available in the Nature Research Reporting Summary linked to this article.

## Supplementary information

Supplementary Information

Reporting Summary

## Data Availability

The soil respiration and microbial biomarker data that support the findings of this study are available in figshare^77^ with the identifier 10.6084/m9.figshare.14130065.
