# Supplementary material for: Adaptation to chronic drought modifies soil microbial community responses to phytohormones
Source: Commun Biol. 2021 May 3;4:516. doi: 10.1038/s42003-021-02037-w (PMC8093232; doi:10.1038/s42003-021-02037-w)
Supplement: Supplementary file 1 — Supplementary Information [file 42003_2021_2037_MOESM1_ESM.pdf]

SUPPLEMENTARY MATERIALS

Sayer *et al.* Adaptation to chronic drought modifies soil microbial community responses to phytohormones

Supplementary Methods 1 - Calculation of carbon inputs and outputs

We calculated total carbon (C) inputs for the 25 μl aliquots of phytohormone solution added to each microplate well, based on the molecular mass of C and the molar concentrations† of the solutions (1 mM, 1μM, and 1nM). When the increase in soil respiration with phytohormone addition was significant ($p < 0.05$) or marginally significant ($p < 0.1$), we calculated the amount of extra C respired in response to phytohormone inputs based on the increase in total CO_2-C relative to the procedural controls for each treatment.

At the highest phytohormone dose (1 mM), the extra C respired was generally equivalent to or less than the C input with phytohormones, although the extra respired C in response to JA and ABA in irrigated plots was slightly higher than the total input. However, at intermediate and low doses of phytohormones, the extra C respired exceeded inputs by several orders of magnitude (Table S1). At the intermediate dose (1 μM) the increase in soil respiration was only significant for ABA addition in droughted and irrigated plots but exceeded C inputs more than 100-fold in both cases. At the lowest dose (1 nM), the extra C respired exceeded inputs by more than a factor of 10^5 with ACC addition in all soils and with JA addition in droughted and irrigated soils; respiration did not increase in response to ABA addition at the lowest dose (Table S1).

Table S1. Comparison of carbon (C) inputs and outputs in response to additions of three phytohormones to soils from long-term control, drought, and irrigated plots during a 6-h microplate assay. Carbon inputs were calculated based on the molecular mass of C† and are shown for 25 μl additions of phytohormone solution at three different concentrations (Mol L^{-1}) to microplate wells containing 0.35 g soil, where ABA is abscisic acid, ACC is 1-aminocyclopropane-1-carboxylic acid, and JA is jasmonic acid. The extra C respired is shown for significant or marginal increases ($p < 0.1$) in respiration in response to phytohormone additions compared to procedural controls.

		**C input**	**Extra C respired** (μg per well)		
**Dose**	**Hormone**	(μg per well)	**Control**	**Irrigated**	**Drought**
1 mM	ABA	4.5	0.97 ±0.43	4.54 ±1.08	1.70 ±0.42
	ACC	3.6	-	1.08 ±0.36	0.76 ±0.11
	JA	1.2	-	1.56 ±0.17	-
1 μM	ABA	0.0045	-	1.27 ±0.38	0.50 ±0.17
	ACC	0.0036	-	-	-
	JA	0.0012	-	-	-
1 nM	ABA	4.5×10^{-6}	-	-	-
	ACC	3.6×10^{-6}	0.46 ±0.26	0.88±0.39	0.81±0.49
	JA	1.2×10^{-6}	-	0.99±0.35	0.94±0.49
Root exudate solution		232.7			

† Abscisic acid: $C_{15}H_{20}O_4$; 1-aminocyclopropane-1-carboxylic acid: $C_4H_7NO_2$; Jasmonic acid: $C_{12}H_{18}O_3$

Supplementary Methods 2 – Combined addition of phytohormones and root exudate solution

To evaluate the influence of phytohormones as constituents of root exudates, we assessed changes in soil respiration in response to adding individual phytohormones in isolation (main text) and in conjunction with a standard root exudate solution (henceforth RE solution), based on 13 published studies of root exudate composition[1] (Table S2). The RE solution was prepared according to existing protocols[1] and divided into ten aliquots, nine of which received added ABA (RE_{ABA}), ACC (RE_{ACC}) and JA (RE_{JA}) in concentrations of 1 mM, 1 μM and 1 nM and the tenth was applied as RE solution without added hormones; we also included procedural controls with deionised water (dH_2O). Three analytical replicates for each sample and treatment were prepared in microplates and measured as described in the main text.

Table S2. Chemical composition of the root exudate solution added to soils from chronic climate change treatments; showing the inputs of carbon (C) and nitrogen (N) per litre of solution; modified from Lopez-Sangil et al. (2017)[1]

Compound/ concentration	mg L^{-1}	% mass	% mol	mg C L^{-1}	mg N L^{-1}
D-glucose	544	30.0%	29.5%	217.6	0
Sucrose	544	30.0%	15.5%	229.0	0
Total carbohydrates		***60.0%***	***45.0%***	***446.6***	***0***
Ammonium oxalate	228	12.6%	15.7%	38.5	45.0
Sodium acetate	153	8.4%	18.2%	44.8	0
Disodium succinate	110	6.1%	6.6%	32.6	0
Ammonium citrate	88	4.9%	3.8%	28.0	10.9
Fumaric acid	55	3.0%	4.6%	22.8	0
Total organic acids		***35.0%***	***49.0%***	***166.7***	***55.9***
L-glutamic acid	57	3.1%	3.0%	18.3	4.3
L-proline	18	1.0%	1.5%	9.4	2.2
L-serine	16	0.9%	1.5%	5.5	2.1
Total amino acids		***5.0%***	***6.0%***	***33.2***	***8.6***

We first evaluated the influence of the standard RE solution on respiration rates among climate treatments by comparing against the procedural controls using linear mixed effects models (*lmer* function in the lme4 package[2] in R version 3.4.0[3]) with RE addition, climate and their interaction as fixed effects, and block as a random effect. We subsequently compared the effects of standard RE solution to the effects of RE solution with phytohormones at different concentrations (RE_{ABA}, RE_{ACC}, and RE_{JA}) using linear mixed effects models with climate, phytohormone, concentration and their interactions as fixed effects, and block as a random effect, whereby the standard RE solution represented 0 M phytohormone concentration. All LMEs were simplified by sequential removal of terms, comparing models with AIC and *p*-values. The best models were compared to appropriate null models using likelihood ratio tests and the final model fit was assessed with diagnostic plots[4].

There was no clear influence of RE solution on soil respiration and no difference among climate treatments in response to the addition of RE solution alone. However, the general patterns of increased respiration with the addition of phytohormones persisted when they were added in conjunction with RE solution (climate + concentration; $\chi^2 = 15.87$, $p = 0.007$; Figure S1). Greater variation in respiration rates with

added RE solution attenuated the effects of phytohormone addition, but we nonetheless observed significant increases in respiration rates in response to the highest concentrations of phytohormones (1 mM; $p = 0.0021$) compared to the standard RE solution, and the effects were strongest in the droughted soils ($p = 0.022$).

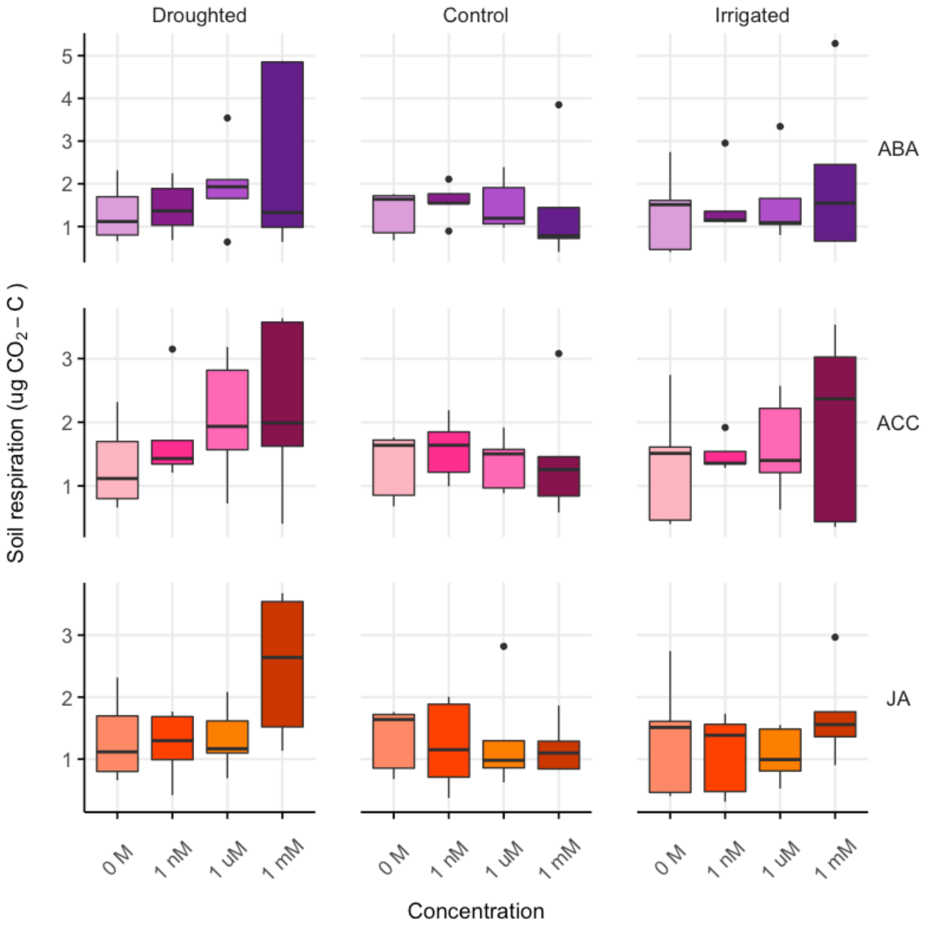

Figure S1. Soil respiration rates (CO_2 efflux) following the addition of root exudate (RE) solutions containing phytohormones at three concentrations, compared to RE solution without phytohormones (concentration = 0 M), where ABA is abscisic acid, ACC is 1-aminocyclopropane-1-carboxylic acid and JA is jasmonic acid; the soils were collected from control, droughted and irrigated plots in a long-term climate change experiment; boxes denote the 25th and 75th percentiles and median lines are given for $n = 5$, whiskers indicate values up to 1.5× the interquartile range, and dots indicate outliers.

Supplementary Methods 3 - Multivariate analysis of pre-incubation microbial communities

We assessed the differentiation of microbial communities among climate treatments prior to our incubation experiments with multivariate analyses, using the vegan package[5] in R version 3.4.0[3]. To assess shifts in soil microbial communities among climate treatments, we used non-metric multidimensional scaling (NMDS) based on Bray-Curtis dissimilarities of the relative abundances of PLFA biomarkers (*MetaMDS* function). A stable two-dimensional solution (stress score = 0.105, R^2 = 0.99) was achieved. The effects of climate treatment on microbial community composition were subsequently examined by permutational multivariate analysis of variance (PerMANOVA; *adonis* function), using 999 permutations constrained within replicate blocks to generate significance values.

The chronic climate treatments at Buxton resulted in divergent soil microbial communities at the plot level (PerMANOVA $F_{2,12}$ = 3.79, R^2 = 0.39, p = 0.001). The NMDS ordination of soil microbial communities clearly separated the drought plots from the control and irrigated plots along the second ordination axis, with minor overlap between control and irrigated plots along the first ordination axis (Figure S2).

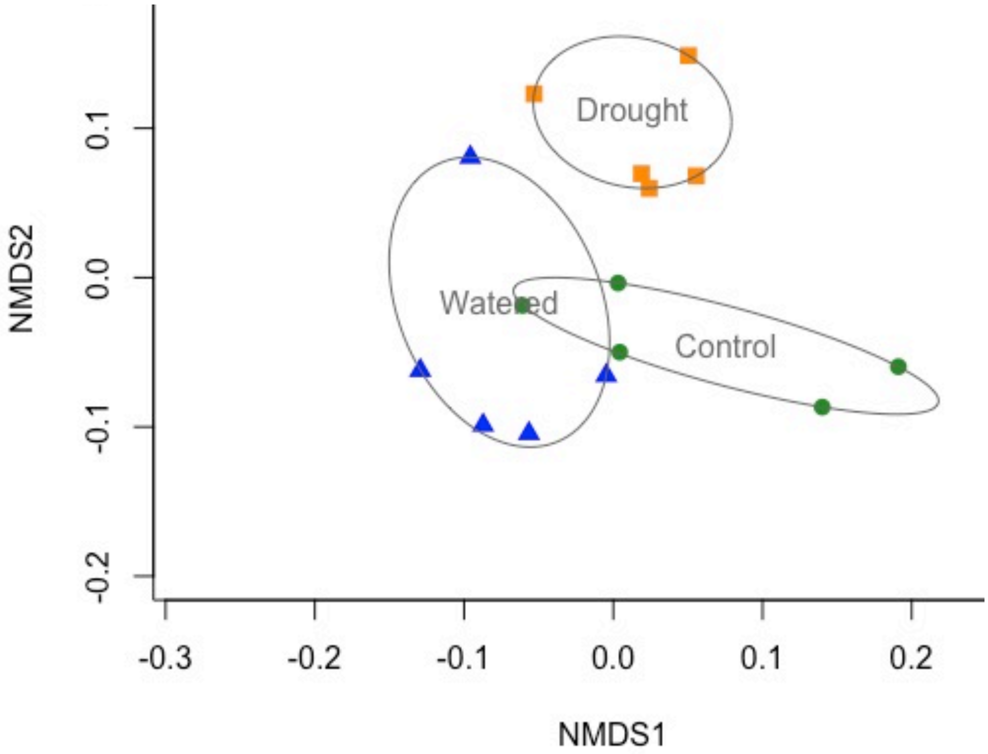

Figure S2. Nonmetric multi-dimensional scaling (NMDS) representation of the soil microbial communities in long-term climate change treatments; showing five replicate control (green dots), drought (orange squares) and irrigated plots (blue triangles), characterised by phospholipid fatty acid biomarkers; ordinations were based on Bray-Curtis dissimilarities and ellipses represent standard errors for climate treatments based on 99% confidence intervals.

Supplementary Methods 4 - Additional lab trials

Two separate trials assessed the repeatability of results for our phytohormone addition experiment. Both trials used soil samples from the same climate treatment plots at Buxton but collected at different times (October 2018 and May 2019).

Trial 1: Pilot test of the microplate assay

The first test was a pilot run of the MicroResp™ incubations[6], using the same soil quantities, hormone solutions and concentrations as described in the main text. The pilot test used soils from the drought (DT) and control (CT) climate treatments only, which were collected from the field in October 2018. The experiment comprised five replicates each of the two climate treatments and 10 hormone treatments (3 hormones at 3 concentrations + procedural controls) in a factorial design without analytical replicates, making 100 micro-incubations in total. Respiration was measured after 6 h and 24 h of incubation as described in the main text and corrected using the mean of 18 blank wells.

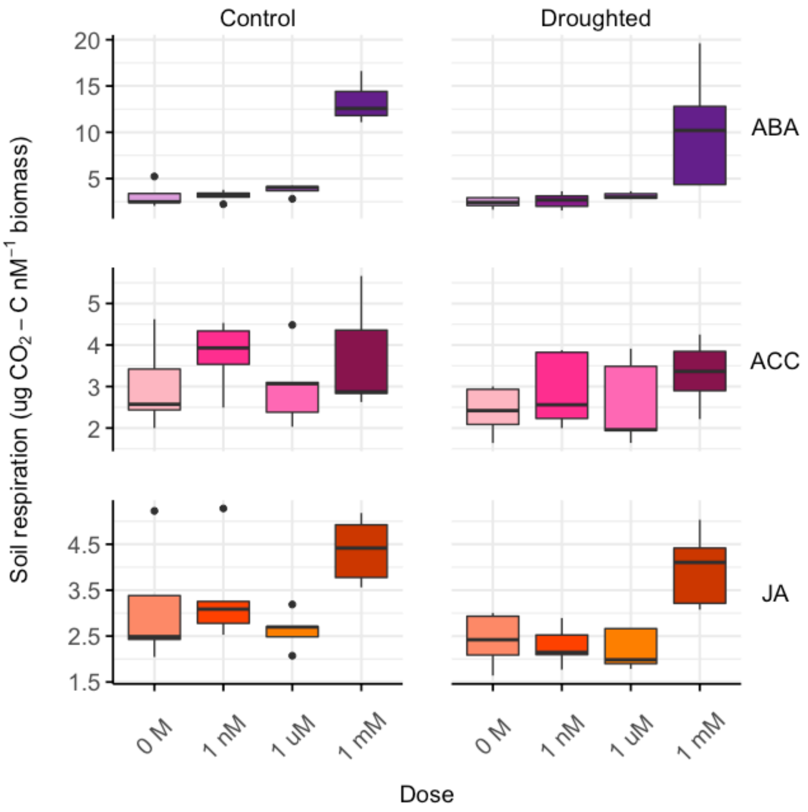

Figure S3 Mean mass-specific respiration (CO_2 efflux per unit microbial biomass) in response to phytohormone additions at three different concentrations compared to procedural controls (concentration = 0), where ABA is abscisic acid (purple), ACC is 1-aminocyclopropane-1-carboxylic acid (pink) and JA is jasmonic acid (orange); the soils were collected from replicate control and droughted plots in a long-term climate change experiment; abbreviations and symbols follow the legend for Figure S1.

Soil respiration (CO_2 efflux) was standardised by unit active microbial biomass (total PLFA biomass) in untreated field soils. The effects of climate and hormone dose on CO_2 released during the incubations were

analysed for each hormone separately using linear mixed effects models (*lmer* function in the lme4 package[2]) in R version 3.4.0[3] with climate treatment, hormone concentration and their interaction as fixed effects, and block and time as a random effect.

Soil respiration increased following addition of all three hormones (Figure S3). Soil respiration responses to lower doses of phytohormones were more apparent after 24 hours of incubation than at 6 hours (Figure S4). Despite lower overall respiration responses in the droughted soils (Figure S3), there were no significant interactions between climate treatments and hormone concentrations. Nonetheless, respiration increased following addition of ABA (χ^2 = 70.6, p < 0.001), ACC (χ^2 = 12.2, p = 0.016), and JA (χ^2 = 43.2, p < 0.001), and the general patterns for ABA and ACC were remarkably similar to those measured in the main incubation experiment (Figures 1 and S3).

After adding 1 mM ABA, respiration increased four-fold in both soils (t = 9.00, p < 0.001) but there was no effect of ABA at any other concentration (Figures S3, S4a). After adding 1 mM ACC respiration increased by 11% in the control soils and 21% in the droughted soils (t = 2.31, p = 0.024). Respiration tended to increase in response to 1 nM ACC (25% in controls and 23% in droughted soils; t = 1.83, p = 0.072), whereas 1 µM ACC had no effect (Figures S3, S4b). After adding 1 mM JA, respiration increased by 41% in the controls and 64% in droughted soils (t = 5.11, p < 0.001), but there was no effect of JA at any other concentration (Figures S3, S4c).

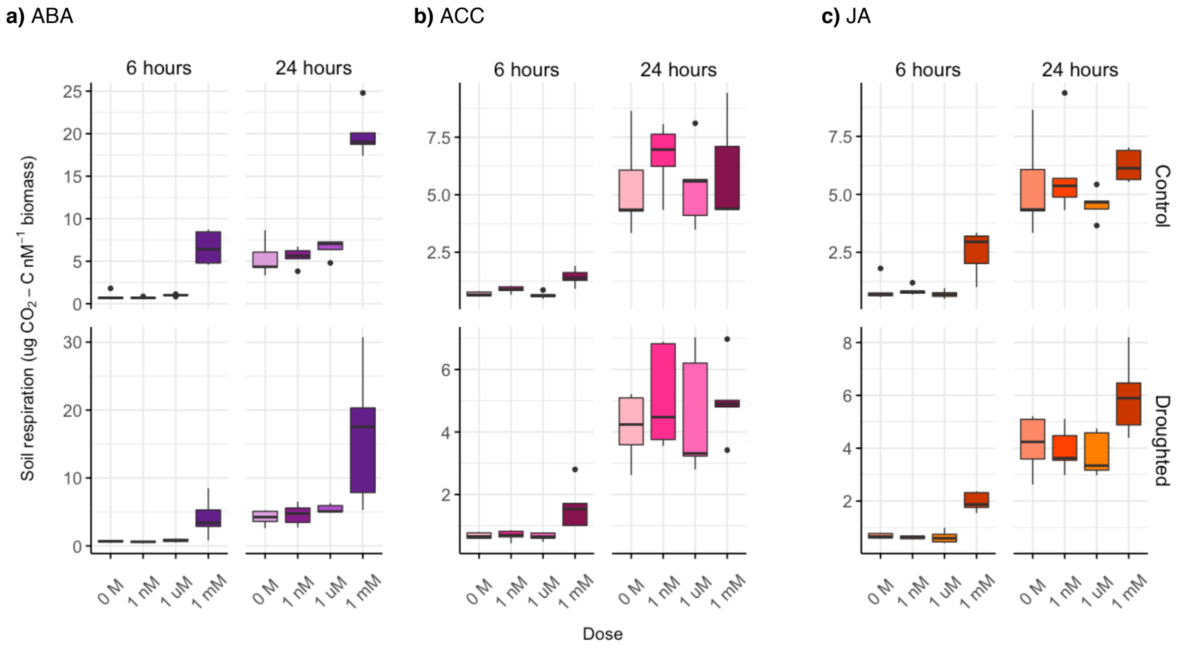

Figure S4 Changes in specific respiration (CO_2 efflux per unit microbial biomass) in control (top panels) and droughted (bottom panels) soils in response to additions of **a)** ABA, **b)** ACC, and **c)** JA at three different concentrations, compared to procedural controls (concentration = 0) after 6 and 24 hours of incubation; abbreviations and symbols follow the legend for Figure S1.

Trial 2: Microcosm experiment - The second test assessed whether using a greater mass of soil (14× the mass of the microplate method) and direct measurements of CO_2 efflux gave consistent results. Samples were collected from the control, droughted and irrigated treatment plots at Buxton in May 2019 and 5 g of fresh soil were added to 50-ml incubation jars. The soils were amended with 360 µl of dH_2O (procedural controls) or 1 mM hormone solution. Hence the microcosm experiment comprised five replicates each of four hormone treatments (dH_2O, ABA, ACC, JA) and three climate treatments in a factorial design, making 60 microcosms in total. Soil CO_2 efflux was measured immediately before/after the addition of hormones, and again after 3, 9 and 24 hours of incubation at 20°C, using a multiplexed infrared gas analyser adapted for laboratory incubations (Li-8100/Li-8150; LiCor Biosciences, Lincoln NE, USA). Each measurement lasted 1 minute with a 30-s pre-purge interval and a 10-s dead-band to flush the tubes and mix the air in the headspace. Soil respiration (CO_2 efflux) was standardised by unit active microbial biomass (total PLFA biomass) in untreated field soils. We analysed the effects of climate treatment and hormone addition on CO_2 efflux rates using linear mixed effects models with climate treatment, hormone treatment and their interaction as fixed effects, and block and time as random effects.

The microcosm incubations also clearly demonstrated that hormone addition affected soil respiration over time in all climate treatments. Soil respiration was generally higher with hormone addition relative to procedural controls (χ^2 = 23.9, p < 0.001) and the increase was particularly strong for ABA addition (t = 3.27, p = 0.001). After 24 hours, soil respiration was 32% higher in the control soils and two-fold higher in droughted and irrigated soils with ABA addition compared to procedural controls (Figure S5).

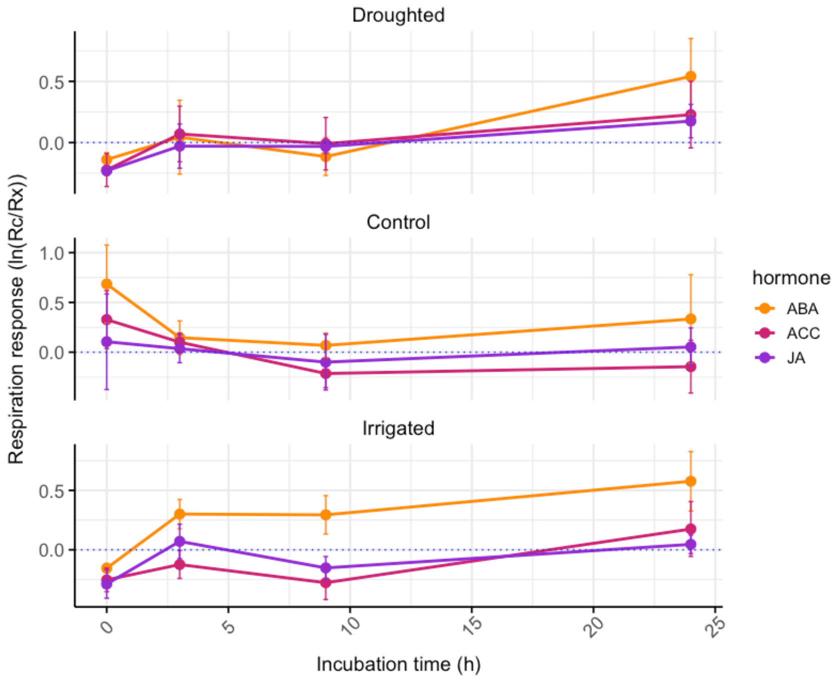

Figure S5 Respiration response of soils from long-term drought, control and irrigated treatments following 1 mM additions of ABA, ACC, and JA during a 24-hour incubation; log response ratios are given relative to procedural controls (dotted blue line) abbreviations follow the legend for Figure S1.

Supplementary Methods 5 - Classification of Phospho-lipid Fatty Acids

As there are very few truly specific phospho-lipid fatty acid (PLFA) biomarkers[7], we used groups of PLFA biomarkers that are likely to be more reliable indicators than individual signature PLFAs[8] (Table S3). As PLFAs are essential components of microbial membranes, the groups also represent differences in the composition and stability of cell walls that are relevant to the stress-tolerance of organisms[9]. Hence, we created functional biomarker groups based on widely accepted primary origins of PLFAs, while acknowledging that many PLFAs will also be found in lower quantities in other organisms[7,10]. We followed broad classifications[8,10,11], using branched chain fatty acids (iso, anteiso) as indicators for Gram-positive bacteria, monounsaturated, hydroxy, and cyclopropyl fatty acids for Gram-negative bacteria[8,12,13], and 10-methyl fatty acids for Actinomycetes[9]. As many peaks overlap among organisms, our analyses of changes in biomarker functional groups are based on the relative proportions of biomarkers, expressed as a percentage of the total PLFAs (mol%) in a given sample.

Table S3. Phospholipid fatty acid biomarkers used to represent microbial functional groups in soils from chronic climate change treatments. Nomenclature follows Bartelt-Ryser et al. (2005)[11] and AM fungi is arbuscular mycrorrhizal fungi.

Functional group	Peaks			
AM fungi	16:1 w5c			
Saprophytic fungi	18:2 w6c	18:1 w9c		
Gram-negative bacteria	10:0 2OH	10:0 3OH	12:1 w8c	12:1 w5c
	13:1 w5c	13:1 w4c	13:1 w3c	12:0 2OH
	14:1 w9c	14:1 w8c	14:1 w7c	14:1 w5c
	15:1 w9c	15:1 w8c	15:1 w7c	15:1 w6c
	15:1 w5c	14:0 2OH	16:1 w9c	16:1 w7c
	16:1 w6c	16:1 w4c	16:1 w3c	17:1 w9c
	17:1 w8c	17:1 w7c	17:1 w6c	17:1 w5c
	17:1 w4c	17:1 w3c	16:0 2OH	17:0 cyclo w7c
	18:1 w8c	18:1 w7c	18:1 w6c	18:0 cyclo w6c
	18:1 w3c	19:1 w9c	19:1 w8c	18:1 w5c
	19:1 w6c	19:0 cyclo w9c	19:0 cyclo w7c	9:1 w17c
	20:1 w9c	20:1 w8c	20:1 w6c	19:0 cyclo w6c
	20:1 w4c	20:0 cyclo w6c	21:1 w9c	21:1 w8c
	21:1 w6c	21:1 w5c	21:1 w4c	21:1 w3c
	22:1 w9c	22:1 w8c	22:1 w6c	22:1 w5c
	22:1 w3c	22:0 cyclo w6c	24:1 w9c	24:1 w7c
	11:0 iso 3OH	14:0 iso 3OH		
Gram-positive bacteria	11:0 iso	11:0 anteiso	12:0 iso	12:0 anteiso
	13:0 iso	13:0 anteiso	14:1 iso w7c	14:0 iso
	14:0 anteiso	15:1 iso w9c	15:1 iso w6c	15:1 anteiso w9c
	15:0 iso	15:0 anteiso	16:0 iso	16:0 anteiso
	17:1 iso w9c	17:0 iso	17:0 anteiso	18:0 iso
	17:1 anteiso w9c	17:1 anteiso w7c		
	19:0 iso	19:0 anteiso	20:0 iso	22:0 iso
Actinomycetes	16:0 10-methyl	17:1 w7c 10-methyl	17:0 10-methyl	18:1 w7c 10-methyl
	18:0 10-methyl	19:1 w7c 10-methyl	22:0 10-methyl	20:0 10-methyl

Supplementary Methods 6 – Phytohormones influence mass-specific respiration rates

As initial microbial biomass differed among climate treatments (Table 1), we also evaluated the influence of phytohormone additions on soil CO_2 efflux expressed as the specific respiration rate per unit microbial biomass (R_{MASS}) to indicate differences in microbial metabolic activity[14]. The effects of ABA, ACC and JA addition R_{MASS} were assessed by separate linear mixed effects models (*lmer* function in the lme4 package[2]) in R version 3.4.0[3] with climate treatment, concentration and their interaction as fixed effects, and block as a random effect. Model selection was achieved by sequentially dropping terms and comparing models using AIC and *p*-values. The final models were compared to corresponding null models using likelihood ratio tests[4].

The respiration rate (R_{MASS}) of all soils increased following phytohormone addition but there was no clear trend in respiration responses with increasing phytohormone concentration (Figure S6). However, the magnitude of respiration responses to phytohormone addition differed among climate treatments, whereby the changes in R_{MASS} were generally greater in irrigated soils compared to droughted or control soils (Figure S6).

Following ABA addition, R_{MASS} increased only at the highest concentration (1 mM) and the increase differed among climate treatments (climate x concentration interaction: $\chi^2 = 48.7$, $p < 0.001$; Figure S6), with a significantly greater respiration response in the irrigated soils than the controls ($p < 0.001$) and droughted soils ($p = 0.001$). Although the increase in R_{MASS} from the control soils ($d = 0.8$) was not significant ($p = 0.13$), R_{MASS} in droughted soils doubled in response to the highest concentration of ABA ($d = 1.95$; $p = 0.015$) and increased more than five-fold in the irrigated soils ($d = 2.27$; p < 0.001). There was a marginally significant trend towards a two-fold increase in R_{MASS} in irrigated soils at the intermediate concentration of ABA (1 uM; $p = 0.085$). Hence, the respiration response to ABA was strongest in irrigated soil.

ACC addition also stimulated R_{MASS}, and the response at different concentrations was similar among treatments (climate + concentration effect; $\chi^2 = 15.84$, $p = 0.007$; Figure S6). R_{MASS} increased at ACC concentrations of 1 mM ($p = 0.02$) and 1 nM ($p = 0.03$), but not at 1 μM. The increase in R_{MASS} from the control soils in response to ACC was negligible ($d < 0.3$), whereas R_{MASS} from droughted soils was 1.5x higher than the procedural controls ($d = 1.42$ and $d = 0.80$ for 1 mM and 1 nM, respectively), and R_{MASS} from the irrigated soils was twice as high ($d = 1.10$ and $d = 0.99$ for 1 mM and 1 nM, respectively). Thus, soil respiration showed an unusual response to ACC addition, which was more pronounced in irrigated soil.

Adding JA had only small, marginally significant effects on R_{MASS}, which were unaffected by climate treatment (concentration effect; $\chi^2 = 7.43$, $p = 0.06$; Figure S6). Surprisingly, the lowest concentration of JA (1 nM) had the largest effect on R_{MASS} ($p = 0.01$, $d = 0.5$, 0.1 and 0.11 for control, droughted and irrigated soils, respectively), whereas the increase in R_{MASS} at the highest concentration was only apparent in the irrigated soils (1 mM, $p = 0.08$, $d = 3.1$) and there was no effect at the intermediate concentration of JA (1 μM). Thus, the lowest concentration of JA stimulated R_{MASS} regardless of climate treatment.

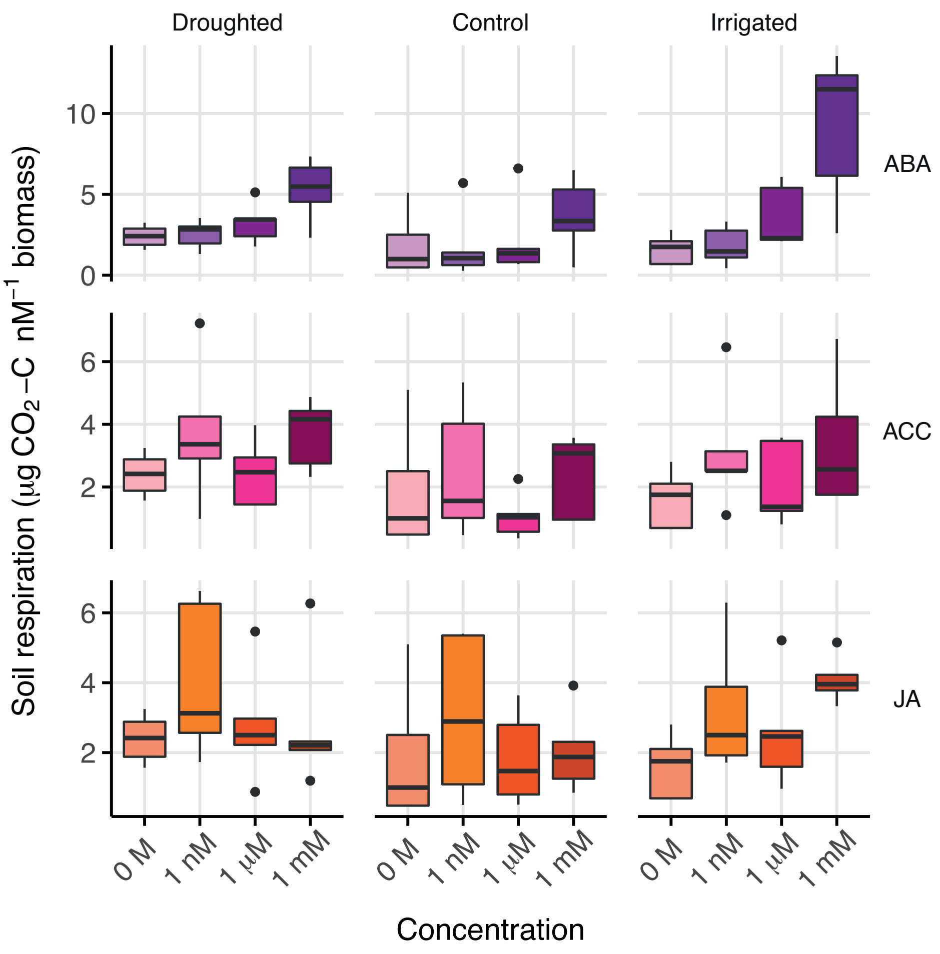

Figure S6. Specific respiration rates (R_{MASS} as CO_2 efflux per unit microbial biomass) following phytohormone additions at three concentrations to soils from long-term climate change treatments compared to procedural controls (concentration = 0 M), where ABA is abscisic acid, ACC is 1-aminocyclopropane-1-carboxylic acid and JA is jasmonic acid; the soils were collected from control, droughted and irrigated plots in a long-term climate change experiment; abbreviations and symbols follow the legend for Figure S1.

Supplementary References

1. Lopez-Sangil, L. *et al.* (2017). The Automated Root Exudate System (ARES): a method to apply solutes at regular intervals to soils in the field. *Methods in Ecology and Evolution*, 8, 1042-1050.
2. Bates, D.M., Mächler, M., Bolker, B., & Walker, S. (2014). Fitting Linear Mixed- Effects Models using lme4. *Journal of Statistical Software*, 67, 1–48.
3. R Core Team (2019) R: A language and environment for statistical computing. R Foundation for Statistical Computing, Vienna, Austria. URL https://www.R-project.org/.
4. Pinheiro, J.C., & Bates, D.M. (2000) *Mixed-Effects Models in S and S-PLUS.* Springer New York.
5. Oksanen, J. *et al.* (2011) vegan: Community Ecology Package. R package version 1.17-11.
6. Campbell, C. D. *et al.* (2003). A rapid microtiter plate method to measure carbon dioxide evolved from carbon substrate amendments so as to determine the physiological profiles of soil microbial communities by using whole soil. *Applied and Environmental Microbiology*, *69*, 3593-3599.
7. Frostegård, Å., Tunlid, A., & Bååth, E. (2011). Use and misuse of PLFA measurements in soils. *Soil Biology and Biochemistry*, 43, 1621-1625.
8. Zelles, L. (1999). Fatty acid patterns of phospholipids and lipopolysaccharides in the characterisation of microbial communities in soil: a review. *Biology and Fertility of Soils,* 29, 111-129.
9. Kaur, A., Chaudhary, A., Kaur, A., Choudhary, R., & Kaushik, R. (2005). Phospholipid fatty acid – a bioindicator of environment monitoring and assessment in soil ecosystem. *Current Science*, 89, 1103-1112.
10. Ruess, L., & Chamberlain, P. M. (2010). The fat that matters: soil food web analysis using fatty acids and their carbon stable isotope signature. *Soil Biology and Biochemistry*, 42, 1898-1910.
11. Bartelt-Ryser, J., Joshi, J., Schmid, B., Brandl, H., & Balser, T. (2005). Soil feedbacks of plant diversity on soil microbial communities and subsequent plant growth. *Perspectives in Plant Ecology, Evolution and Systematics*, 7, 27-49.
12. Frostegård, Å, Bååth, E., Tunlid, A., (1993). Shifts in the structure of soil microbial communities in limed forests as revealed by phospholipid fatty acid analysis. *Soil Biology and Biochemistry*, 25, 723–730.
13. Kerger, B.D. *et al.* (1986). Signature fatty acids in the polar lipids of acid-producing Thiobacillus spp.: methoxy, cyclopropyl, alpha-hydroxy-cyclopropyl, branched and normal monoenoic fatty acids. *FEMS Microbiology Ecology* 38, 67e77.
14. Anderson, T. H., & Domsch, A. K. (1993). The metabolic quotient for CO_2 (qCO_2) as a specific activity parameter to assess the effects of environmental conditions, such as pH, on the microbial biomass of forest soils. *Soil Biology & Biochemistry*, 25, 393-395.
