# Supplementary material for: Adaptation to chronic drought modifies soil microbial community responses to phytohormones
Source: Commun Biol. 2021 May 3;4:516. doi: 10.1038/s42003-021-02037-w (PMC8093232; doi:10.1038/s42003-021-02037-w)
Supplement: Supplementary file 2 — Reporting Summary [file 42003_2021_2037_MOESM2_ESM.pdf]

nature research

Corresponding author(s): E.J.Sayer

Last updated by author(s): Feb 27, 2021

Reporting Summary

Nature Research wishes to improve the reproducibility of the work that we publish. This form provides structure for consistency and transparency in reporting. For further information on Nature Research policies, see our Editorial Policies and the Editorial Policy Checklist.

Statistics

For all statistical analyses, confirm that the following items are present in the figure legend, table legend, main text, or Methods section.

n/a	Confirmed	
☐	☒	The exact sample size (n) for each experimental group/condition, given as a discrete number and unit of measurement
☐	☒	A statement on whether measurements were taken from distinct samples or whether the same sample was measured repeatedly
☐	☒	The statistical test(s) used AND whether they are one- or two-sided<br>*Only common tests should be described solely by name; describe more complex techniques in the Methods section.*
☐	☒	A description of all covariates tested
☐	☒	A description of any assumptions or corrections, such as tests of normality and adjustment for multiple comparisons
☐	☒	A full description of the statistical parameters including central tendency (e.g. means) or other basic estimates (e.g. regression coefficient) AND variation (e.g. standard deviation) or associated estimates of uncertainty (e.g. confidence intervals)
☐	☒	For null hypothesis testing, the test statistic (e.g. F, t, r) with confidence intervals, effect sizes, degrees of freedom and P value noted<br>*Give P values as exact values whenever suitable.*
☒	☐	For Bayesian analysis, information on the choice of priors and Markov chain Monte Carlo settings
☐	☒	For hierarchical and complex designs, identification of the appropriate level for tests and full reporting of outcomes
☐	☒	Estimates of effect sizes (e.g. Cohen's d, Pearson's r), indicating how they were calculated

Our web collection on statistics for biologists contains articles on many of the points above.

Software and code

Policy information about availability of computer code

Data collection	No software was used for data collection
Data analysis	R version 3.4.0. was used for all analyses

For manuscripts utilizing custom algorithms or software that are central to the research but not yet described in published literature, software must be made available to editors and reviewers. We strongly encourage code deposition in a community repository (e.g. GitHub). See the Nature Research guidelines for submitting code & software for further information.

Data

Policy information about availability of data

All manuscripts must include a data availability statement. This statement should provide the following information, where applicable:

- Accession codes, unique identifiers, or web links for publicly available datasets
- A list of figures that have associated raw data
- A description of any restrictions on data availability

The manuscript includes the following data availability statement: The soil respiration and microbial biomarker data that support the findings of this study are available in figshare with the identifier DOI 10.6084/m9.figshare.14130065

Field-specific reporting

Ecological, evolutionary & environmental sciences study design

All studies must disclose on these points even when the disclosure is negative.

Study description	The Buxton Climate Change Impacts Study is located on calcareous grassland in Derbyshire, UK. The climate treatments have been applied to 3-m × 3-m plots in five fully randomized blocks since 1993. We sampled soils from three climate treatments: summer drought, in which rainfall is excluded using automated shelters from July-August ('droughted'); supplemented rainfall to 20% above the long-term average from June-September ('irrigated'); and control plots that experience the ambient climate. The samples were subsequently used in laboratory assays, which retained the original experimental design (5 replicates per climate treatment, based on samples collected from replicate field plots) but included up to 10 phytohormone addition treatments (3 hormones x 3 concentrations + 1 procedural control to account for water added to the soils) in a fully factorial design.
Research sample	Bulk soil samples collected at 0-10 cm depth, homogenised and roots removed
Sampling strategy	The sampling strategy was determined by the design of the field experiment: 5 plots of three treatments assigned to replicate blocks. The laboratory studies retained the design of the field experiment, such that any analytical replicates were pooled to give means for 5 replicates per treatment.
Data collection	Soil samples were collected by co-authors Crawford and Edgerley; data from the soil analyses were collected by co-author Crawford, and a commercial laboratory using appropriately standardised and calibrated laboratory instruments, described in full in the methods section. All data were collated into csv files and checked by lead author Sayer before analysis.
Timing and spatial scale	Samples were collected in October 2018, when soil water content in the droughted plots had recovered to control levels. Three samples were collected at random locations within each 3-m x 3-m experimental plot (one plot per treatment and replicate block). The cores were taken at 0-10 cm depth using a 1-cm diameter punch corer and then bulked to give one sample per plot.
Data exclusions	We did not exclude any data from the study.
Reproducibility	All analyses and experiments are reproducible, and the majority of our laboratory analyses were carried out in duplicate. Tests to verify methodology and support interpretation are described in the supplementary material. All of our methods follow standard published protocols and any modifications are described in the methods.
Randomization	The field experimental treatments (climate) were applied to randomly assigned plots within replicate blocks; the phytohormone treatments were applied randomly to subsamples within each climate treatments.
Blinding	The field experiment includes control treatments, the laboratory experiments include procedural controls, and all laboratory analyses included appropriate blanks.

Did the study involve field work? ☒ Yes ☐ No

Field work, collection and transport

Field conditions	Samples were collected from limestone grassland in October 2018, when soil water content in the droughted plots had recovered to control levels. Samples were stored in cooler boxes for transport and refrigerated or frozen within 8 hours of collection. The cores were sieved (2-mm) to remove stones and debris and stored at 4ºC for five days before subsequent laboratory assays under controlled conditions.
Location	The study site is located within the grounds of the Health and Safety Executive at Harpur Hill, Buxton, Derbyshire, UK. The vegetation is ancient limestone grassland.
Access & import/export	We have full approval to access the site from the Health and Safety Executive, UK and the Steering Committee of the Buxton Climate Change Impacts Lab. All necessary permits were obtained to export samples to the commercial laboratory in the USA, and remaining material was destroyed after analysis according to the conditions of the US import permit.
Disturbance	Beyond the immediate effects of the climate treatments, all efforts have been made to limit disturbance to the experimental plots and organisms by taking small and infrequent samples, using non-destructive methods, and removable or biodegradable markers, etc.

Reporting for specific materials, systems and methods

We require information from authors about some types of materials, experimental systems and methods used in many studies. Here, indicate whether each material, system or method listed is relevant to your study. If you are not sure if a list item applies to your research, read the appropriate section before selecting a response.

Materials & experimental systems

n/a	Involved in the study
☒	☐ Antibodies
☒	☐ Eukaryotic cell lines
☒	☐ Palaeontology and archaeology
☒	☐ Animals and other organisms
☒	☐ Human research participants
☒	☐ Clinical data
☒	☐ Dual use research of concern

Methods

n/a	Involved in the study
☒	☐ ChIP-seq
☒	☐ Flow cytometry
☒	☐ MRI-based neuroimaging
